# Tetanus1Read before the Medical Society of the County of Cattaraugus, Nov. 14, 1905.

**Published:** 1906-01

**Authors:** E. M. Coss

**Affiliations:** Cattaraugus, N. Y.


### Tetanus.[1]

By E. M. COSS, M. D., Cattaraugus, N. Y.

As I have had two cases of tetanus following vaccination, I have chosen for my subject that much dreaded and usually fatal disease. Tetanus, as you all know, has been recognised as an incurable disease since the time of Hippocrates, especially in acute cases, though subacute and chronic cases have occasionally recovered. But little has been added to our knowledge concerning it until quite recently. As lately as 1899 I find recorded in Sajous's work, that it is now generally conceded that the conclusions of Rose are, in the main, correct—namely, that tetanus may be produced by any unusual impression upon the nervous

1. Read before the Medical Society of the County of Cattaraugus, Nov. 14, 1905.

system, be that influence of a mechanical, chemical, thermal, or pathalogical character. The principle cause was thought to be any contused or lacerated wound that involved the nerves, more especially of the hands and feet. It was also claimed that atmospheric influences, such as chilliness or dampness, might produce it, and that under certain conditions it might arise idiopathically, the latter due chiefly to exposure to cold. But more recently, due to the investigations of Nicolier in 1884, it was found that tetanus could be produced by injecting garden earth beneath the skin.

I think it is now conceded by all that the disease is due to the tetanus bacillus which was isolated by Kitasato in 1889. This produces a poison or toxin that must enter the circulation. That the point of entrance is more often the seat of a contused or lacerated wound, where the vitality or resisting power of the cell is destroyed, is a fact, for seldom is an incised wound the seat of infection. In fact we find that any abrasion of the skin under favorable circumstances may be the seat of infection. In certain localities the disease is more prevalent than in others; especially is it more frequent in the tropics. The tetanus bacillus is hard to destroy. It may retain its power of development for months or years. Its habitat is the soil, especially garden soil, or soil that has been fertilised with horse manure. It has been found on the walls and floors of rooms in which patients have had the disease; also in horse barns where horses have had tetanus. The dust of the air has been found to contain the germ in places where tetanus is prevalent. In a recent journal I saw reported, by Dr. Smith of Louisiana, that a man became infected with tetanus by being bitten by fleas, while drawing manure from a horse barn,—there being no other sign of an abrasion of the skin or of wounds on his person.

The period of incubation is from three to twenty days, usually less than ten. It is said the longer the symptoms are delayed, the milder the case. Possibly some persons in whom the symptoms are delayed are cases of secondary infection. The germ is confined to the secretions of the wound and its vicinity; at least, they have not been found circulating in the blood. We are, therefore, led to believe that the symptoms are due to a toxin produced by the bacilli at the point of infection. Here is an interesting feature of the case. It has been proved by recent investigators, Marie and Morax, 1902, and Meyer and Ransom, 1903, that the transmission of the toxin depends upon the integrity of the axis cylinder of the motor nerves, through the end apparatus or abrasion of which it must enter to be carried to the cerebrospinal centers. It can only travel centripetally and not centrifugally. It seems to have a selective affinity for the motor centers, at which point it is difficult to neutralise the poison or overcome its deadly effect. In the brain and spinal cord minute hemorrhages and distended capillaries have been discovered, showing the nerve centers to be the seat of lesions, though the nerves in the vicinity of the wound have been found to be swollen and injected.

Before referring to the treatment of tetanus, I wish to report two cases following vaccination, which occuurred in my practice, and then three others, to show the latest plan of treatment. My first case occurred in August, 1902.—A bright, healthy boy, eleven years of age, large and well-developed, parents American, mother a very nervous person, had been vaccinated with vaccine virus from a reliable house, about eighteen days, when he began to lose interest in his play, and was not as active as usual, though he did not complain much. This was Thursday; Saturday he rode five miles and back on horseback to his grandfather's and also went to a ball game and lay on the ground during its progress. Sunday he was at his uncle's at dinner and complained that he could not swallow. Monday he was worse and complained that his back was stiff, and he was restless, trismus being pronounced. His symptoms were attributed to horseback riding and to taking cold while lying on the ground at the ball game. In the afternoon the nurse took his temperature and found it below normal, which alarmed her. I was called about 3:30 P. M. and found him unable to open his mouth more than half an inch. His back and neck were quite rigid. That night he began to have convulsions and grew rapidly worse and opisthotonos developed. I began the use of tetanus antitoxin as soon as I could procure it. That was Tuesday, about noon, and I continued to use it until he died, using in all about 200cc. Tuesday the spasms were more frequent and opisthotonos was continuous. Every spasm was accompanied by a groan, which once heard would never be forgotten. The face would become cyanosed with each convulsion and great drops of sweat would stand on his face. Temperature was 101° and pulse 130. Wednesday he became unconscious and had retention of urine. Thursday the convulsions were more severe. I gave chloroform frequently to relieve the spasms, but with little effect. The temperature and the pulse went up to 104° and 140 respectively. Coma deepened and he died about 7 P. M. At the last the spasms reached the anterior parts of the body, so that they would open the mouth instead of closing it. The vaccination sore did not appear any more inflamed than usual, though it was covered with a dirty looking grey crust.

The second case was that of a German girl, fifteen years of age, phlegmatic temperament, vaccinated January 25, 1904, with capillary tubes of glycinised lymph, also made by a trustworthy firm. I washed the arm with boiled water and soap, and sterilised my knife before vaccinating her and covered the sore with a shield. The vaccination took, and pursued a normal course till February 19th, twenty-five days after vaccination. Then I was called as she complained of stiffness of the jaws and neck. I found her with temperature 99½°, and a pulse above 100. She could not open her mouth more than half an inch and the neck was very rigid. That afternoon she had been hanging up some clothes out of doors and had had a spasm of the jaws severe enough so that she bit her tongue. There was no tetanus antitoxin in town, so I gave her Baccilli's phenol solution, a dram hypodermically every two hours. The next morning at 9:00 A. M., I gave 20cc. of Mulford's antitetanic serum. No more could be obtained until evening. At that time a nurse came and the following is copied from her report:

Patient very rigid, unable to move without help; temperature 101° F.; pulse 126; respiration 28. Administered 10 cc. of P.D's serum. at 5:30 P. M. Within three hours from the first injection in the evening her temperature fell to 99½°; pulse 112; respiration 22. Temperature remained below 100 till the sixth day, when it went up to 102° in the afternoon; pulse 130. It reached this point for four days, then gradually declined to normal on the eleventh day; pulse 110 to 115. Gave the serum every three hours till the sixth day, when it was given five times, on the seventh day six times, eighth day five times, ninth day twice, tenth day twice, eleventh day three times, twelfth day three times, thirteenth day three times, fourteenth day twice, fifteenth day twice, sixteenth day twice, seventeenth day twice and the last. Gave 720cc. in all, of the antitoxin, continued the phenol solution till the seventh day. Liquid diet was given throughout the disease. Rigidity of the muscles increased and contraction of the spinal muscles till opisthotonos was so great that two or more pillows were kept under the back for support. There was twitching of the muscles and light spasms every few minutes with occasional severe convulsions. On the fifth day occurred the most severe convulsion, lasting for twenty-five minutes. After the fifth day she gradually improved, the spasms being less frequent and lighter. She could move her lower extremities better and about the eleventh day the muscles of the neck began to relax. From then on, improvement was rapid. She was able to sit up one hour on the eighteenth day of her illness, on the nineteenth, two or three hours. In one week more she was able to ride four miles to her home. She had been down stairs and helped about the work several days before this. There was a little stiffness of the muscles of mastication, that is, she could not open her mouth to the full extent for about a week after she went home, though she experienced no difficulty in eating. I gave as a sedative bromo chloral compound, which acted very nicely. The sore on the arm was cleansed and cauterised with silver nitrate, then dusted with an antiseptic powder. I injected the serum in the region of the buttocks. No unpleasant symptoms followed the use of the serum except a slight urticaria, which soon disappeared.

In The Medical Record, May 21, 1904, is the report of a case by Dr. J. H. Rogers, Jr., which I will briefly report in order to show the latest and by some claimed to be the only rational plan of treatment, there being only one case previously reported, as being treated according to this method, and that by Ransom and Meyer.

"J. H., a boy of twelve years, received on March 17th, a gunshot wound of the palm of the left hand, which was dressed at a dispensary. Nothing wrong was noticed till April 1st, when the mother observed that he ate with difficulty. April 2d he was not so well; the 3d the boy was worse, so that he had to lie down. In the afternoon he became rigid and opisthotonos developed. At 11 P. M., under an anesthetic, the brachial plexus was exposed and injections of antitoxin were made in five different nerves, using about 5 to 10 m. to each nerve; and about 130 m. were injected into the spinal canal of the lumbar region after withdrawing and inserting the needle several times in order to cause some abrasion of the motor nerves. The boy was much improved the next day. There still being evidence of tetanus, the operation was repeated. On April 5th, nearly all the rigidity had disappeared, though there was still some stiffness about the neck and masseters. He could chew only with considerable effort. He sat up on the 14th and began to walk on the 18th, and left the hospital, eating well, on the 23d., twenty days after the first injection. No paralysis or bad symptoms followed this treatment."

His second case is in The Medical Record, July 2, 1904. "A boy eleven years old, on Monday, May 2d, seven days after receiving a punctured wound in the foot, from a rusty nail, complained of stiffness of the jaw. He was given 20cc. of antitoxin, hypodermically, which was repeated in the afternoon. On the 3d he was treated by the intraneural and intraspinal method. On the 4th he was worse and the case seemed almost hopeless. Gave a drachm and a half of antitoxin between the second and third dorsal vertebrae. On the morning of the 5th it was found that no convulsion had occurred. No serum was given this day, but on reviewing the case, it was discovered that in making the previous injections into the nerves I overlooked the obturator through which the poison was allowed to flow, which nearly caused the death of the patient, the channels in the anterior crural and sciatic being entirely blocked. May 6th trismus had returned. He being worse, a drachm and a half of antitoxin was injected in the lumbar region of the spinal cord subdurally. May 7th this was repeated. No more was given subsequently. Trismus did not disappear till the 13th. On the 18th he was out of bed and entirely cured, sixteen days after treatment was begun." In this case the antitoxin treatment was begun the first day of his illness.

In the Medical Record, October 15, 1904, page 616, there is an interesting and instructive article by W. Scott Schley, of New York. In that article he reports the case of a boy five years of age, who stuck a sliver in his leg on the outer side of the right knee, June 11th. Symptoms of tetanus began on the 18th; the 19th he was worse and the 20th he had several convulsions, when he was given antitoxin injections, it being injected in the anterior crural and sciatic nerves, 3cc. in each; also the same in the lumbar region of the spinal cord; 10cc was also injected hypodermatically. This was repeated several times. There was one severe spasm on the 25th; after this the spasms decreased in severity and frequency, so that he was allowed to get up July 9th, being twenty days after his admission to the hospital.

He used 176 cc. of serum. My patient sat up on the nineteenth day after I was called and the eighteenth day after treatment was begun." Dr. Schley says the wounds healed kindly, but the advocates of the intraneural and intraspinal treatment as a rule say nothing about paralysis and atrophy of the muscles which sometimes follow this treatment.

The first patient whose case I reported was in the habit of washing his arm with water from the faucet, using his hand. He was caring for a horse at the time and playing with the boys, going in bathing. He frequently removed the shield to show them his arm. The second patient, from seven to ten days before she was taken sick, on several cccasions, after preparing the potatoes for dinner, got some water and bathed the arm around the sore. There was a scab on the sore which was detached on one side so that a drop of water may have found its way under the scab, carrying with it some tetanus germs from her fingers. These germs may have adhered to the fingers after handling the potatoes. I believe these were cases of secondary infection, as we vaccinated from four hundred to five hundred people that winter and this was the only case of tetanus in the lot. The treatment has been given in the description of the cases.

To any one familiar with the recent literature concerning tetanus it must be evident that the serum treatment is curative in many cases that would be fatal under any other form of treatment. It is easy to say, in disputing this statement, that it was a subacute or chronic case and would have recovered under any plan of treatment. My first case must have been acute and the second one was nearly as severe as the first, at the time I first saw it. Statistics show that the mortality has been reduced from 60 to 30% by the serum treatment. That some of the cases which have been treated by the intraneural and intraspinal method have shown brilliant results there can be no question. It may possibly prove to be the best method of treatment, especially the intraspinal injections. I believe, however, that if antitetanus serum is used hypodermatically and in sufficient quantities, we may save many cases that would otherwise prove fatal. That the injection of the nerves of the limb affected with a wound will intercept the major part of the poison, I do not believe. It has been proven that the toxin travels through the motor nerves and the antitoxin takes the same route to the nervous centers, but that it is first carried through the general circulation and taken up by all the motor nerves, I think, is also proven by the fact that, no matter where the infected wound is located, the first symptoms are manifest in the muscles of mastication or in the neck, these being the muscles supplied by the shortest nerves, through which it takes less time to travel than through those which go to the extremities; from here it extends downward gradually as the nerves increase in length. I would suggest that physicians take more pains to instruct their patients to observe antiseptic precautions in caring for their vaccination sores, when they need caring for, or to call at the office for treatment. In conclusion, I would recommend that every physician see that the druggist or health officer has a sufficient quantity of antitetanic serum on hand to treat a case till more can be obtained. If it is your first case, you are apt to be decieved into thinking that your patient is not very sick, especially if it is in the early stages, but you had better make haste with your antitoxin or you will regret it. Therefore have a supply of fresh, reliable serum within reach at all times.

---

Chemist—(to poor woman)—You must take this medicine three times a day after meals.

Patient—But, sir, I seldom get meals these 'ard times.

Chemist—(passing to next customer)—Then take it before.—*Glasgow Times.*

